# Generation and comparative genomics of synthetic dengue viruses

**DOI:** 10.1186/s12859-018-2132-3

**Published:** 2018-05-08

**Authors:** Eli Goz, Yael Tsalenchuck, Rony Oren Benaroya, Zohar Zafrir, Shimshi Atar, Tahel Altman, Justin Julander, Tamir Tuller

**Affiliations:** 1 Department of Biomedical Engineering, Tel Aviv University, Tel Aviv, Israel; 2 SynVaccineLtd. Ramat Hachayal, Tel Aviv, Israel; 3 Institute for Antiviral Research, Utah State University, Logan, UT USA; 4 Sagol School of Neuroscience, Tel Aviv University, Tel Aviv, Israel

**Keywords:** Synthetic virology, RNA viruses, Viral evolution, Flavivirus, Viral comparative genomics, Silent and non-synonymous mutations

## Abstract

**Background:**

Synthetic virology is an important multidisciplinary scientific field, with emerging applications in biotechnology and medicine, aiming at developing methods to generate and engineer synthetic viruses. In particular, many of the RNA viruses, including among others the Dengue and Zika, are widespread pathogens of significant importance to human health. The ability to design and synthesize such viruses may contribute to exploring novel approaches for developing vaccines and virus based therapies.

**Results:**

Here we develop a full multidisciplinary pipeline for generation and analysis of synthetic RNA viruses and specifically apply it to Dengue virus serotype 2 (DENV-2). The major steps of the pipeline include comparative genomics of endogenous and synthetic viral strains. Specifically, we show that although the synthetic DENV-2 viruses were found to have lower nucleotide variability, their phenotype, as reflected in the study of the AG129 mouse model morbidity, RNA levels, and neutralization antibodies, is similar or even more pathogenic in comparison to the wildtype master strain. Additionally, the highly variable positions, identified in the analyzed DENV-2 population, were found to overlap with less conserved homologous positions in Zika virus and other Dengue serotypes. These results may suggest that synthetic DENV-2 could enhance virulence if the correct sequence is selected.

**Conclusions:**

The approach reported in this study can be used to generate and analyze synthetic RNA viruses both on genotypic and on phenotypic level. It could be applied for understanding the functionality and the fitness effects of any set of mutations in viral RNA and for editing RNA viruses for various target applications.

**Electronic supplementary material:**

The online version of this article (10.1186/s12859-018-2132-3) contains supplementary material, which is available to authorized users.

## Background

The ability to synthesize and engineer RNA viruses has important applications to biotechnology and human health [1–5]. Although some previously published studies involved generation and analysis of various synthetic RNA viruses (e.g., refs [6–12]), more studies are required to understand their evolution and population dynamics together with developing efficient synthetic biology approaches for their research [13, 14].

Dengue virus (DENV) is an important mosquito-borne RNA flavivirus with four known genomically diverse serotypes. Its genome is a positive polarity, single stranded RNA of approximately 11 kb composed of three parts: the unique coding sequence (CDS) that produces 10 mature viral proteins via a single precursor polyprotein, and two flanking untranslated regions (UTRs) that contain important structural and functional elements [15–17]. Since dengue is an important human pathogen, widely recognized as a major public health concern [18], new insights into its biological and evolutionary properties including its wildtype and synthetically generated strains may have significant influence on the development of new antiviral vaccines and therapies. The Zika virus (ZIKV), also of the flavivirus genus, is closely related to DENV and was recently classified as a public health emergency of international concern due to recent outbreaks [19, 20]. The DENV and ZIKV genomes encode three structural proteins (capsid, precursor membrane, and envelope) and seven nonstructural proteins (NS1, NS2A, NS2B, NS3, NS4A, NS4B, and NS5) [21, 22].

**Fig. 1:**
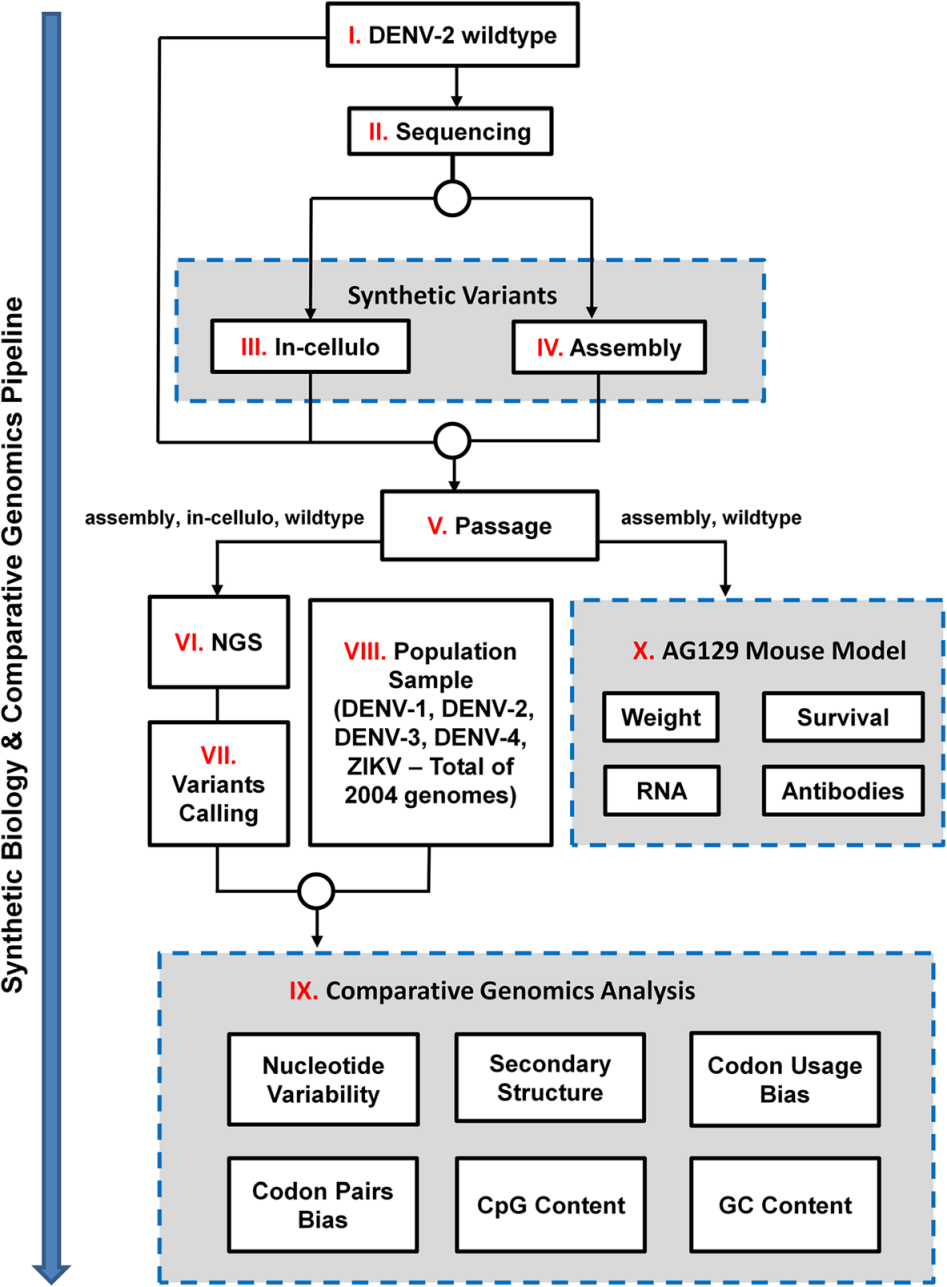
Flow diagram of this study showing the full comparative genomics pipeline developed for synthetic biology of RNA viruses (see details in the main text)

In this work we demonstrate, for the first time, a comprehensive analysis of DENV-2 (Dengue virus, serotype 2) synthetic variants using a multidisciplinary pipeline that combines comparative genomics, synthetic biology, next generation sequencing, and experiments with animal models of viral infection. Specifically, based on DENV-2 New Guinea C wildtype strain (WT; see Methods), two synthetic variants were generated; comparative genomics to other DENV serotypes and to ZIKV, as well as their replication and pathogenesis analyses in AG129 mouse model were performed (see illustration in Fig. 1). The suggested approach can be also directly applied to other RNA viruses.

## Results

### Study outline

The full comparative genomics pipeline for synthetic biology of RNA viruses demonstrated in this study is described in Fig. 1 (see Methods for more details): DENV-2 New Guinea C wild type viral strain (Fig. 1 I) was sequenced (Sanger; Fig. 1 II; see also Additional file 1, section 8). The resulting sequence was used as a master sequence to generate synthetic variants. In order to demonstrate the robustness of the reported results two different approaches were examined: (1) *in-cellulo* construction of subgenomic infectious amplicons (Fig. 1 III), and (2) PCR *assembly* of full-length infectious DNA (Fig. 1 IV). After three passages of the wildtype and synthetic DENV-2 viruses in Vero E6 cells (Fig. 1 V), viral RNA was extracted and next generation sequencing was performed (NGS; Fig. 1 VI). The resulting sequences were verified and analysis of genomic variants was performed, with respect to single nucleotide variations (SNV) and inserts (Variants Calling; Fig. 1 VII). The results were compared to a population sample of 2004 available DENV-1/2/3/4 and ZIKV genomes (see details in Methods; Fig. 1 VIII-IV) with respect to several genomic features: nucleotide variability, secondary structure, codon and codon pair bias, CpG and GC contents. In addition, the wildtype and synthetic viruses (generated by the *assembly* method) were compared in vivo in the AG129 mouse model (Fig. 1 X); the comparison included: weight change, survival rates, RNA levels, and neutralizing antibodies (Ab). In the following subsections we describe the results of our analyses.

### Next generation sequencing and comparative genomics analysis of the synthetic and wildtype viruses

**Fig. 2:**
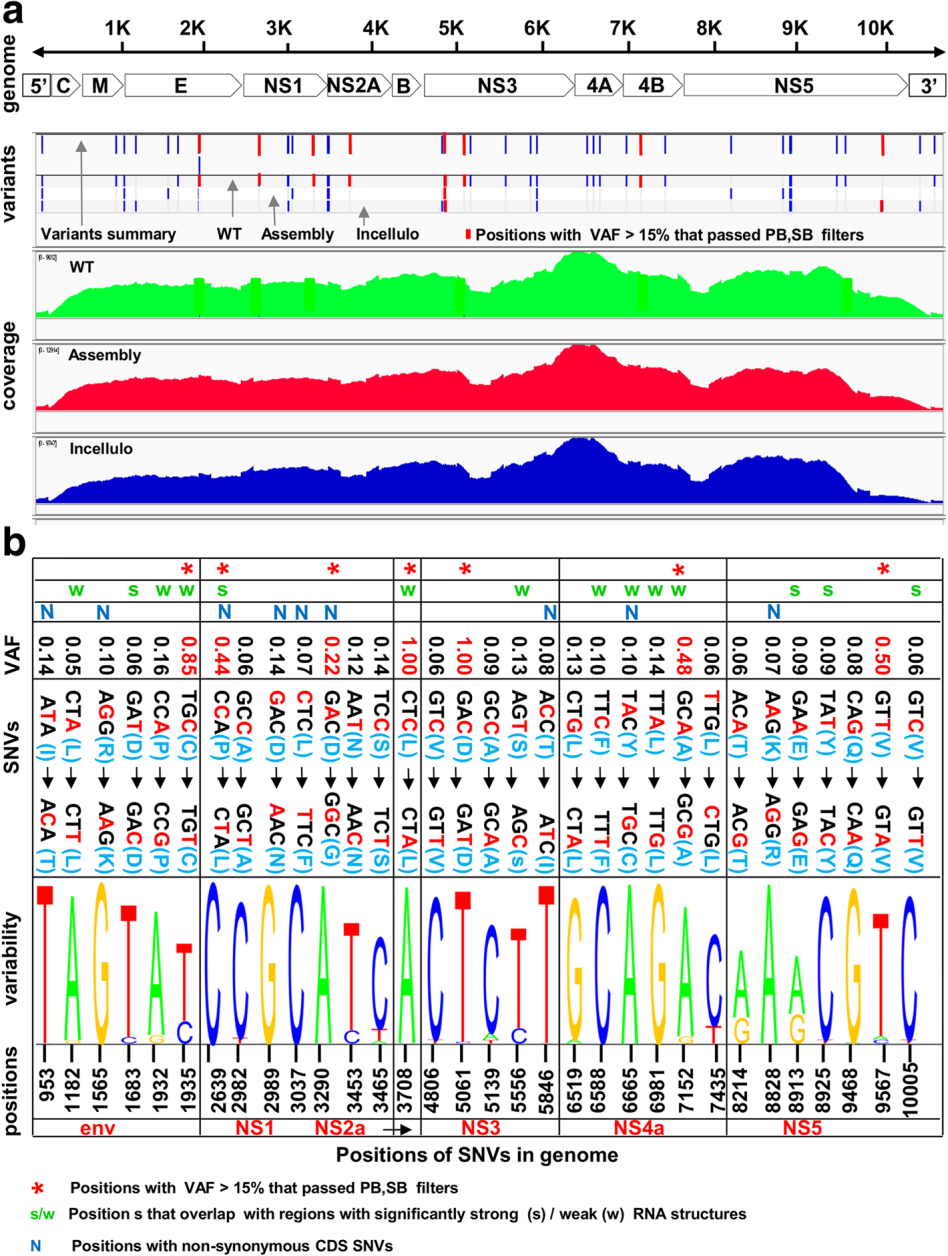
Results of NGS study. **a** Positions of variants (SNVs and inserts) and read coverage along the genome of the wildtype virus (WT) and its synthetic constructs (assembly and *in-cellulo*). Genomic coordinates and annotations are specified in the “genome” panel. Positions of variants are marked by vertical bars (blue and red) in the “variants” panel; summary of variants for all samples (first row in the “variants” panel), and sample-specific variants (three different rows for *in-cellulo*, assembly, and WT) are shown. Eight Positions (1 indel and 7 SNVs) with VAF at least 0.15 that also passed Position Bias and Strand Bias filters were marked by red bars; 6 of them are found in the WT strain only. **b** Analysis of 32 CDS SNVs. The nucleotide variability at SNV positions is based on a set of 618 aligned DENV2 genomes, represented by the corresponding sequence logo in the “variability” panel. The coordinates of SNVs are specified in the x-axis; corresponding genes are specified in the bottom row. The variability at each position is represented by a stack of letters. The relative sizes of the letters indicate their frequency in the alignment at specific position, where the total height of the letters depicts the information content of the position in bits. The SNVs at each position are depicted in the “SNVs” panel in the following format: reference codon (reference AA) → variant codon (variant AA). The altered nucleotides are marked in red. The variant allele frequencies (VAF) at each position were specified in the “VAF” panel. Non-synonymous SNVs (18%) were marked by “N”. Positions that overlap with regions that undergo a conserved selection for strong or weak mRNA folding (based on [16]) were marked by “s” or “w” respectively. Seven CDS SNVs with VAF of at least 0.15 that also passed Position Bias and Strand Bias filters were marked by red asterisks

**Fig. 3:**
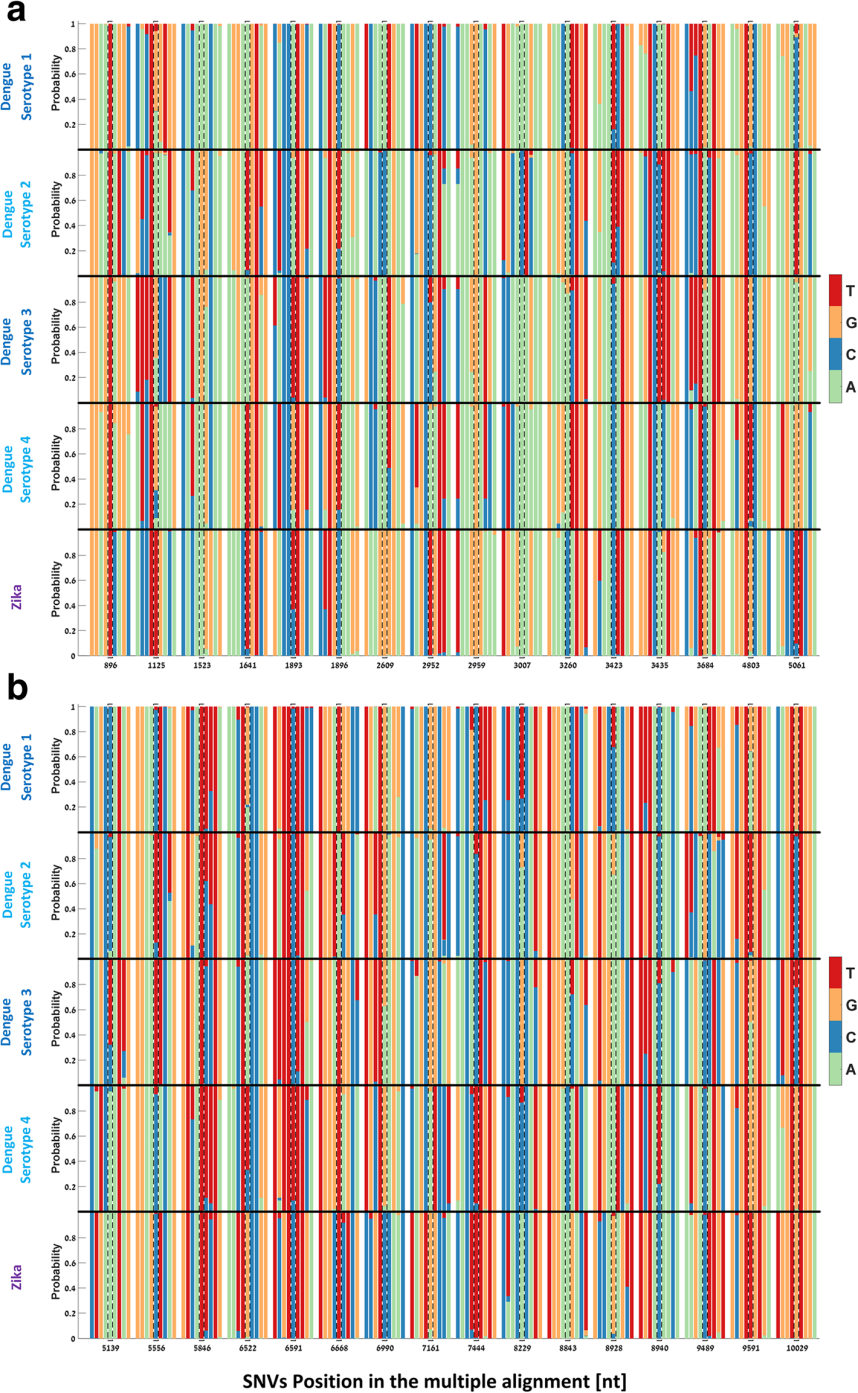
Sequence conservation and the surroundings of the SNVs in the multiple aligned genomes of the DENV serotypes and ZIKV. The nucleotide probability distribution for each SNV (marked in dashed line) and 4 upstream/downstream nucleotides is shown. The nucleotide representation is based on their amino acid sequences; see Methods. **a** Multiple alignments of SNVs 1–16. **b** Multiple alignments of SNVs 17–32

**Fig. 4:**
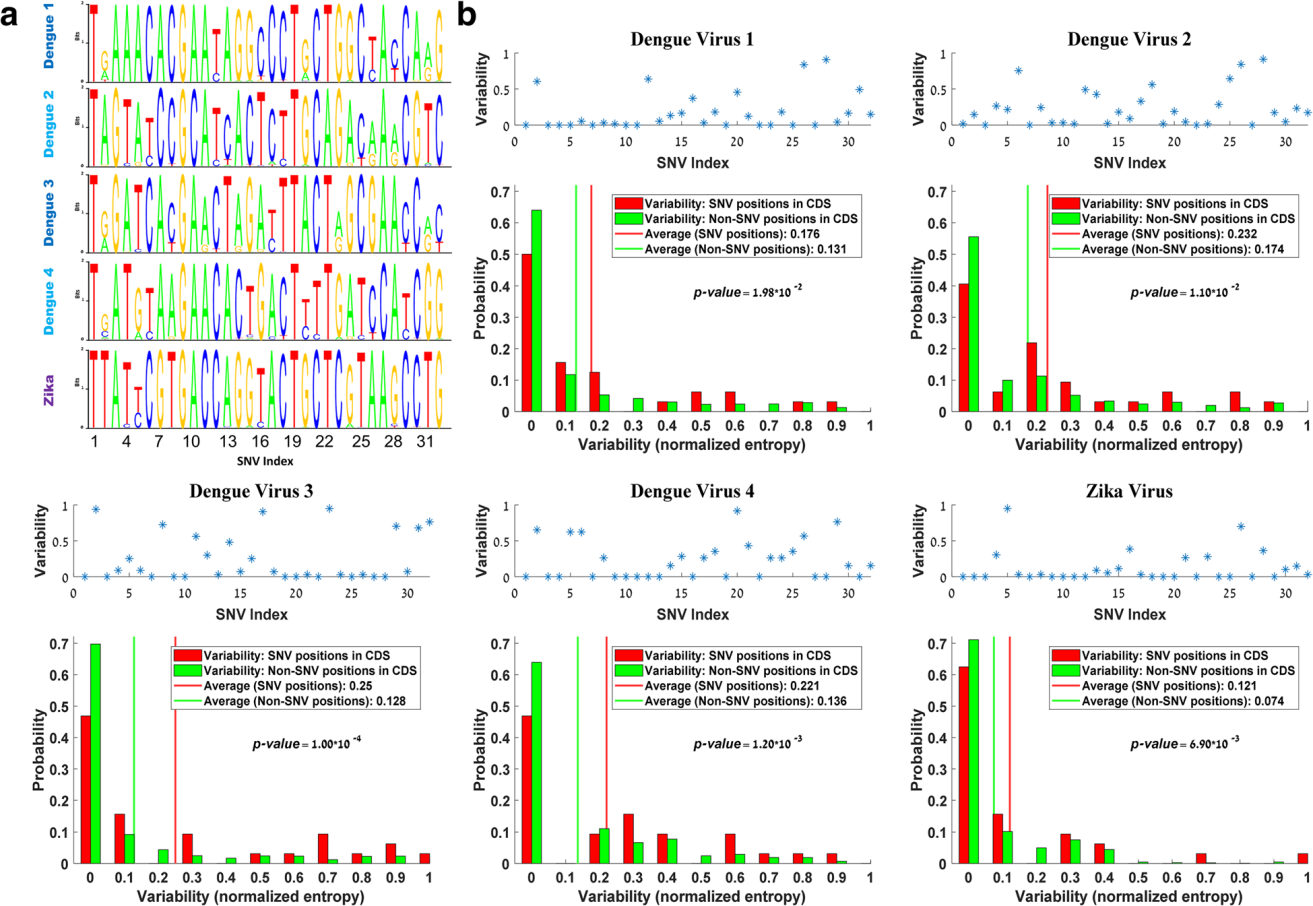
SNV variability analysis. **a** Sequence logo presentation of the SNVs variability in each of the 4 DENV serotype and ZIKV. **b** Variability distributions of the SNV and Non-SNV positions. The Non-SNV values were randomly selected from the 1st/2nd/3rd positions in their codons (relatively to their frequencies), such that the subsets of the Non-SNV has the same position distribution as the SNVs. The results show significantly higher values in the SNV case among the genomes of all DENV serotypes and ZIKV (empirical *p* < 0.02; see details in the Methods)

**Fig. 5:**
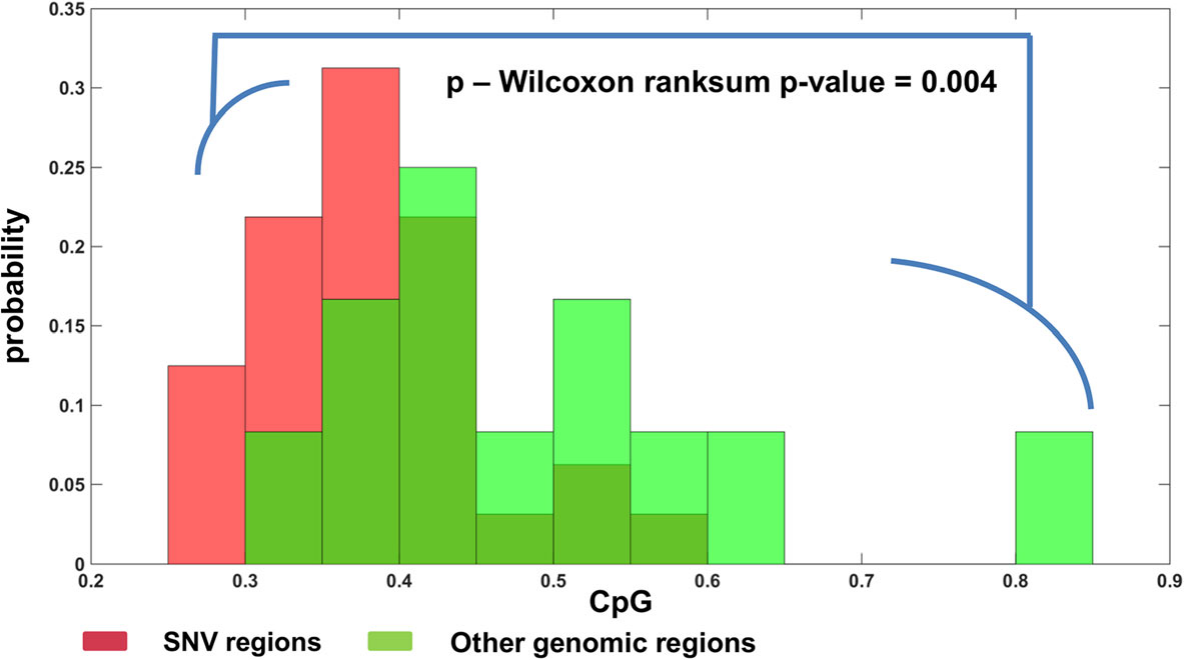
CpG dinucleotides are significantly suppressed in regions of 100 codons around SNVs as compared to genomic regions of the same size that do not contain any SNV

The NGS and comparative genomics analysis of the populations of synthetic and WT viruses in the cell lines is summarized in Figs. 2–5 (further details can be found in the Methods).

The positions of the discovered variants (SNVs and inserts) and read coverage along the genome for the WT virus and its synthetic constructs (*assembly* and *in-cellulo*) are shown in Fig. 2a: Genomic coordinates and annotations are specified in the “genome” panel. In total, 38 variants were found: 35 in the CDS and 3 in the UTRs; positions of these variants were marked by vertical bars (blue and red) in the “variants” panel. 32 (91%) of the variants in the CDS were due to SNVs, and the rest 3 (9%) were due to inserts; 23 (72%) of SNVs in the CDS were synonymous. In the UTRs all variants were due to inserts. All the inserts were due to an additional ‘A’ nucleotide within a stretch of A’s. 64% of all SNVs were found in a WT virus (but not in synthetic variants). Positions of 8 variants (7 SNVs and one insert) with variant allele frequencies (VAF) of at least 15% that also passed various filters (Position Bias [PB] and Strand Bias [SB] filters; see Methods), were marked by red bars (“variants” panel); 6 of them were found in the WT strain only.

Additional analysis of the 32 CDS SNVs is summarized in Fig. 2b: Most of the SNVs were C → T or A → G. As expected, the majority of the variations were found to be synonymous (72%). SNVs at positions 3708 and 5061 are substitutions (VAF = 1), the others (VAF < 1) may indicate polymorphisms within the population (panels “SNVs” and “VAF”). These polymorphisms may be related either to errors in viral replication [23] or to sequencing errors (for very low VAF), and may play a role in improving the fitness of the WT virus [23–25]. Seven significant SNVs (VAF ≥ 15% that also passed PB and SB filters) were marked by red asterisks. Further, the positions of the CDS SNVs were compared to the population sample consisting of 618 available DENV-2 genomes (see Methods for additional details). In particular relations to regions with significantly strong / weak RNA structures ([16] and Methods) and to position-wise nucleotide variability (“variability” panel) were analyzed. Specifically: 14 out of 32 (44%) of SNVs in CDS were found to overlap with regions that undergo a conserved selection for strong or weak mRNA folding, although this overlap is not statistically significant with respect to random (*p*-value = 0.14, see Methods); this result suggests that only some of the SNVs are related to secondary structures and may influence the viral fitness via the effect on the RNA folding. Moreover, no accord between the SNVs and the nucleotide variability at the corresponding positions in the alignment of the population sample (quantified by normalized entropy, see Methods) was found (i.e. SNV’s do not tend to appear in more or less conserved positions). Also, Spearman correlation between variant frequencies derived from the NGS data and the frequencies of these variants observed in the population sample at SNV positions was found to be low/insignificant (rho = 0.16, *p*-value = 0.38). These results may demonstrate that the variability at the population level is a dynamic trait and may differ both at different stages of the replication cycle within the same host and across different hosts.

To check the relation between the variability within the sequenced WT DENV-2 population and within population samples of other DENV serotypes and ZIKV, we mapped the 32 SNV’s positions identified in the CDS to the multiple alignment of combined 2004 genomes of 4 DENV serotypes and ZIKV (652/618/357/45/332 genomes for DENV-1/DENV-2/DENV-3/DENV-4/ZIKV, respectively; see details in Methods) and analyzed their surroundings, as can be seen in Fig. 3. A detailed presentation of the sequence logos of all SNV’s in each of the 4 DENV serotype and ZIKV can be seen in Fig. 4a.

Following, we compared the nucleotide variability in these positions to the variability in the other, non-SNV positions along the CDS. Specifically, since the variability of the 3rd nucleotide in the codon is higher than of the other positions, we performed the analysis of the Non-SNVs positions such that it will have the same distribution as the SNVs; see more details in the Methods. As can be seen in Fig. 4b, the average SNV’s nucleotide variability is higher than the average variability in non SNV positions for all the DENV serotypes and ZIKV (empirical *p*-value < 0.02 for equal size relative sampling; see details in Methods). These results suggest that the SNV’s positions also tend to be more variable in other DENV serotypes and/or in ZIKV.

Finally, we analyzed the association of SNVs with additional local different genomic features, namely: Codons Usage Bias as measured by ENC (Efficient Number of Codons), Codon Pairs Bias (CPB), CpG Content (CpG), and GC Content (GC) (see Methods). Various studies relate these features to different genomic mechanisms, which take part in the viral replication cycle and are related to the viral fitness. For example, it was suggested that viral codons may be under selection to improve the translation efficiency, among others, via adaptation to the host tRNA pool (or other translation resources; e.g., [26–38]). It was also suggested that translation efficiency is affected not only by single codons, but also by distribution of codon pairs (although it is debated whether this feature is under a direct selection or can be a consequence of distribution of dinucleotides [39–42]. In refs [43–45] it was shown that CpG pairs are under-represented in many RNA and most small human DNA viruses, in correspondence to dinucleotide frequencies of their hosts. This phenomenon can be related, for example, to the contribution of the CpG stacking base-pairs to RNA folding [46] and/or to the enhanced innate immune responses to viruses with elevated CpG [47]. The stability of the RNA secondary structures can also be affected by the genomic GC content [48]. By analyzing local genomic regions of 100 codons we found that regions surrounding the positions of SNVs were characterized by a significantly lower CpG content (Wilcoxon rank-sum test, *p*-value = 0.004) compared to regions of the same size that didn’t contain any SNV (Fig. 5). As was explained above, the higher mutation rates in the CpG suppressed regions may be associated with a stronger evolutionary pressure, possibly related to RNA folding or to the interaction with the immune system. However, we did not find any significant differences between the SNV and non-SNV regions with respect to the rest of the analyzed features (see Additional file 1, section 1).

### AG129 mouse model study

**Fig. 6:**
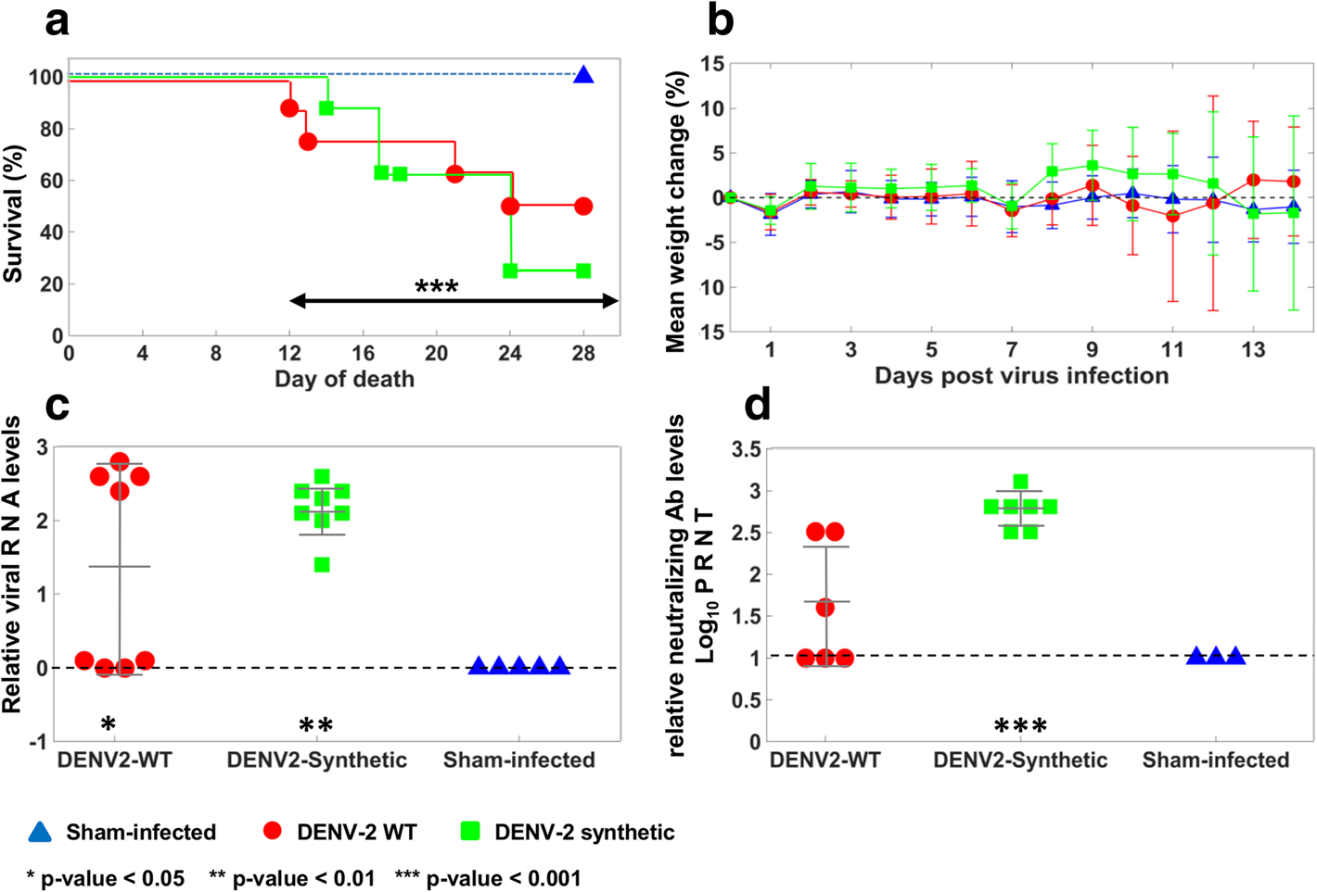
Results of AG129 mouse model study. **a**. Percentage of survival of AG129 mice infected with wild-type and synthetic DENV-2 (*p*-value < 0.001 for 12 dpi and furhter, as compared with sham controls). **b**. Mean weight change (%) of AG129 mice infected with different DENV-2 variants. **c** Virus titers from serum collected 3 dpi from infected AG129 mice (**p-value < 0.01, *p-value < 0.05, as compared with sham controls). **d** 50% plaque reduction neutralization titers (PRNT50) from serum taken 14 dpi (***p-value < 0.001, as compared with sham controls). Note that there were not sufficient quantities of serum for 5 samples: 2 from group 1(infected with WT variant), 1 from group 2 (infected with synthetic variant) and 2 from control group for quantification of neutralizing Ab

An AG129 mouse model was used to characterize the pathogenicity and immunization capabilities of synthetic DENV-2, generated by the *assembly* method. Infection with the WT virus resulted in around 50% mortality (Fig. 6a, Table 1), which was consistent with intraperitoneal infection of AG129 mice in previous studies in our lab. Synthetic wild-type virus injection resulted in 75% mortality and had a similar mortality curve to mice infected with the WT virus. The mortality rates caused by both synthetic and WT infection were significantly higher (*p*-value < 0.001) than in sham-infected controls (Fig. 6a).

**Table 1:** Morbidity and mortality of AG129 mice infected with wild type and synthetic DENV-2 New Guinea C strain

Animals: Male and female AG129 mice			Duration of experiment: 28 days		
Virus route: Intraperitoneal injection			Volume: 0.2 ml		
	Toxicity controls		Infected, treated		
Treatment	Virus titer (ge/μg RNA)^a^	PRNT_50_ titer (GM ± SD)^b^	Alive / total	Virus titer (ge/μg RNA)	PRNT_50_ titer (GM ± SD)^b^
DENV-2 WT	–	–	4/8	1.3 ± 1.4*	1.5 ± 0.7
DENV-2 synth	–	–	2/8	2.1 ± 0.3**	2.8 ± 0.2***
Vehicle control	0.0 ± 0.0	1.0 ± 0.0	–	–	–

*GM* geometric mean, *SD* standard deviation

^a^Virus titer expressed as virus titer equivalents (estimated by CCID50/ml stock) per μg total RNA

^b^mean day to death of mice dying prior to 31 dpi. PRNT_50_–50% plaque reduction neutralization titers

***p-value < 0.001, **p < 0.01, *p < 0.05, as compared with sham-infected [vehicle] controls

Some weight loss was observed at various times over the course of infection in individual animals infected with WT or synthetic DENV-2, although the general trend was for little average weight change beyond baseline within all groups (Fig. 6b). For some reason, vehicle inoculated control animals (6–7 weeks old) did not gain weight as expected (Fig. 6b), although reasons for this are unknown.

Viral RNA was detected in all animals infected with synthetic DENV-2 in serum collected 3 days post infection (dpi), which was significantly (*p*-value < 0.01) higher than sham-infected controls (Fig. 6c). Half of the animals inoculated with the WT virus had detectable titers, while the other half had RNA levels below that of assay detection (Fig. 6c). Despite the lower rate of detection of viral RNA in these samples, the mean viral RNA level in this group was significantly (*p*-value < 0.05) elevated as compared with controls. No viral RNA was detected in the serum of sham-infected mice.

As anticipated, animals infected with synthetic DENV-2 elicited significantly (*p*-value < 0.001) elevated neutralizing Ab levels on 14 dpi as compared with sham-infected controls (Fig. 6d). Infection with WT virus resulted in a variable response, usually including detection of viral RNA and mortality. No neutralizing Ab was observed in sham-infected mice.

## Discussion

This study demonstrates the construction of multidisciplinary pipeline for comparing and analyzing synthetic and wildtype viruses. Central aspects/steps of this pipeline includes comparative genomics: First, synthetic viruses can be designed based on comparative genomics analysis of WT viral genomes (e.g., ref. [16, 49]). Second, the synthetic variants can be analyzed based on comparative genomics tools; this includes the comparisons of the variants to each other and to WT genomes. Among others, this study demonstrates the interactions between comparative genomics and other research tools in synthetic virology. Specifically, we report here for the first time a set of SNVs that appear in a master wildtype DENV-2 virus in comparison to its synthetic constructs. In general, the wildtype virus was found to have a higher genomic variability (i.e. more nucleotide variations) than its synthetic strains. It is known that the population of viral variants is important for its fitness [24, 25]; thus, this analysis demonstrates an approach for connecting viral fitness to the structure of its population.

In addition, our study demonstrates that the synthetic variants may cause more morbidity and mortality than the wildtype virus. Also, infection with the synthetic virus resulted in higher serum viral RNA titers (as measured 3 dpi) and more consistent and high levels of neutralizing Ab (as measured 14 dpi) as compared to infection with the wildtype DENV-2. These findings may be related to the already mentioned fact that the synthetic virus includes lower levels of genomic sequence variability. RNA viruses are often defined as quasispecies [50], collections of closely related mutant spectra or mutant clouds. The fact that a virus exists as a ‘cloud’ of closely related genomes constitutes an evolutionary advantage, among others by enabling the virus to efficiently adjust to different hosts or cells. Therefore, higher level of variability in the wildtype virus may improve its replication rate in hosts such as Vero cells (the origin of the investigated wildtype virus) and/or humans/*A. aegypti*, but on the other hand, decrease its fitness/replication rate in mice. It is also possible that the wildtype virus undergoes a direct selection for reduced virulence (among others this may be related to its variability) which actually increases its transmissibility and fitness [51].

We used both the *assembly* and the *in-cellulo* synthetic constructs for the NGS analysis and variant calling. However, currently due to various constraints, for the mouse model study we only used the synthetic DENV-2, generated by the *assembly* method. It would be interesting in the future to characterize the properties of the *in-cellulo* construct, since the *in-cellulo* and *assembly* viruses have different SNVs which might impact lethality*.*

The approach described here can be performed on any RNA virus and can be used as a platform technology for the rational design and synthesis of live attenuated vaccines for RNA viruses. Specifically, in the near future we plan to use this approach for studying the effect of synonymous mutations on the fitness of various types of viruses (e.g., the other 3 serotypes of dengue and the Zika virus). To this end, we will generate large libraries of viral genomes with different type of synonymous mutations. The RNA and protein levels of these libraries will be measured in cell lines and model organisms and the mutations will be connected to the observed measurements and to other viral phenotypes (e.g. cytopathic effect).

## Conclusions

In this study we presented a multidisciplinary pipeline that combines tools from synthetic biology, next generation sequencing and comparative genomics, for engineering synthesizing and analyzing synthetic RNA viruses. We applied this pipeline to generate synthetic Dengue viruses and compare them to their wildtype master strain. In particular, we showed that although the synthetic Dengue viruses were found to have lower nucleotide variability, their phenotype, as reflected in the study of the AG129 mouse model morbidity, RNA levels, and neutralization antibodies, was similar or even more pathogenic in comparison to the wildtype. The described approach can be performed on any RNA virus and can be used as a platform technology for rational design and synthesis of oncolytic RNA viruses and live attenuated vaccines.

## Methods

### Virology

#### Reagents

Minimum Essential Medium Eagle - Earle’s (EMEM medium), Fetal Bovine Serum (FBS), Penicillin-Streptomycin-Nystatin Solution, and Dulbecco’s Phosphate Buffered Saline (DPBS) were obtained from Biological Industries (Bet-Haemek, IL). Opti-MEM medium was obtained from Gibco-ThermoFisher (Grand Island, NY). TransIT-X2 Mirus reagent was also used (MC-MIR-6003; Madison, WI).

#### Cell lines and virus

Vero E6 (ATCC CRL-1586) cells were cultured in EMEM supplemented with 10% Fetal Calf Serum, streptomycin 0.1 mg/ml, penicillin 100 u/ml, nystatin 12. 5 U/ml, 0.29 mg/ml at 37 °C in a 5% CO_2_ incubator. DENV-2, New Guinea-C strain was prepared in Vero E6 cells grown in EMEM medium containing 2% inactive FBS, Strep-Pen-Nys solution and incubated at 37 °C for 10 to 12 days until the appearance of cytopathic effects (CPE). The supernatant was then harvested and divided into aliquots and stored at − 80 °C. Viral RNA was quantified using RT-PCR and infectious tissue culture assay.

#### Cell transfection and infection

Vero E6 cells 20,000 cell/well were seeded in 96-well tissue culture plates and incubated overnight at 37 °C. A final amount of 2000 ng of an equimolar mix of all cDNA was amplified by PCR and incubated with 2.4 ml of TransIT-X2 Mirus reagent in 400 ml of Opti-MEM medium. Following the manufacturer’s instructions, the mix was incubated for 15 min at room temperature, then divided into 8 wells (of a 96-well plate) with Vero E6 cells and incubated for 4 h. After incubation the cell supernatant was removed and cells were washed three times with D-PBS, and 100 ml of EMEM medium supplemented with 2% inactive FBS was added. The supernatant was harvested 10 to 12 days post transfection. The supernatant was centrifuged and then passaged two more times as follows: 0.8 to 1 ml supernatant was added to 25 cm2 culture flask of Vero E6 cells (400,000 cells incubated overnight), and incubated for 4 h. After incubation the cell supernatant was removed and washed three times with D-PBS, and 6 ml of EMEM medium supplemented with 2% inactive FBS was added. The cell supernatant was incubated for 10 to 12 days until the appearance of cytopathic effects (CPE). The supernatant was then harvested divided to aliquots and stored in at 80 °C.

### Molecular biology

#### Dengue virus sequencing and construction of subgenomic amplicons’ plasmids

Dengue virus type 2, New Guinea C (Culture collection) RNA was extracted from aliquoted culture supernatants using the QIAamp Viral RNA mini kit (Qiagen). The cDNA was synthesized using Maxima H Minus First Strand DNA synthesis kit (Thermo Scientific) with random hexamer primers, according to manufacturer’s instructions. The cDNA was amplified by PCR in 500-3000 bp overlapping amplicons using PfuUltra II Hotstart PCR Master Mix(Agilent Technologies). The 500-1000 bp amplicons were sequenced using the Sanger method. The 2500-3500 bp amplicons were cloned into pJET1.2/blunt vectors using the CloneJET PCR Cloning Kit (Thermo Scientific). Cytomegalovirus promoter was inserted before the first fragment (0–2.5 kb). The last fragment (7–10.7 kb) was synthesized using the solid-phase DNA synthesis method (BioBasic), followed by hepatitis delta and simian polyadenylation signal.

#### Subgenomic infectious amplicons

Dengue subgenomic amplicons were generated as previously described in [6]. Entire viral genome was amplified by PCR in 4 DNA fragments of approximately 3 kb, each with 75-80 bp overlapping arms. The first fragment was flanked at 5′ by the cytomegalovirus promoter. The last fragment was flanked at 3′ by the hepatitis delta ribozyme followed by the simian polyadenylation signal. The PCR products were purified using MinElute PCR purification kit (Qiagen) and 1000 ng of equimolar mix was transfected to Vero E6 cell line (ATCC).

#### Assembly PCR of full-length infectious DNA

Dengue viruses’ genomes were amplified by PCR in 4 DNA fragments of approximately 3 kb, each with 100-500 bp overlapping ends. The first fragment was flanked at 5′ by the cytomegalovirus promoter. The last fragment was flanked at 3′ by the hepatitis delta ribozyme followed by the simian polyadenylation signal. The overlapping fragments were extended using Q5 High-Fidelity DNA Polymerase (New England Biolabs), using the neighboring fragment as a primer.

#### NGS sequencing

Dengue virus type 2, New Guinea C (Culture collection) RNA was extracted from aliquoted culture supernatants using QIAamp Viral RNA mini kit (Qiagen). cDNA was synthesized using Maxima H Minus First Strand DNA synthesis kit (Thermo Scientific) with random hexamer primers, according to manufacturer’s instructions. The cDNA was amplified in seven 1700–2500 bp overlapping amplicons using PfuUltra II Hotstart PCR Master Mix (Agilent Technologies). PCR products were separated in a 1% agarose TAE gel. After confirming the bands of interest were present without non-specific products, the PCR product was purified using the MIniElute PCR purification kit (Qiagen). Sequencing libraries were prepared using an in-house (INCPM) DNA-Seq protocol, and sequenced 2 × 150 on an Illumina MiSeq nano v2.

### Computational analysis

#### NGS analysis and variants calling

Three DENV cDNA samples were sequenced and analyzed: one of the samples was the wildtype (WT) strain, with the WT (Sanger) sequence used as a reference genome for NGS analysis. The two (*assembly* and *in-cellulo*) synthetic variants of the virus were synthesized using two different methods (full length assembly PCR and subgenomic infectious amplicons). The WT sequence can be found in Additional file 1, section 8. Sequencing libraries were prepared using the INCPM DNA-seq protocol, and sequenced 2 × 150 on an Illumina MiSeq nano v2 PE150. Sequenced reads were mapped to a reference genome (genome of the wildtype virus) using BWA MEM v0.75 [52]. Among all read-pairs with the same alignment, a single representative read was chosen using Picard MarkDuplicates (https://broadinstitute.github.io/picard/).

Variants were called using Freebayes v1.0.2 [53] with the setting -f 0.05 --ploidy 5 (in order to increase sensitivity to low allele fraction variants). Freebayes is a haplotype based Bayesian genetic variant detector designed to find small polymorphisms and complex events (composite insertion and substitution events) smaller than the length of a short-read sequencing alignment. It uses short-read alignments (BAM files with Phred+33 encoded quality scores) for individuals from a population and a reference genome to determine the most-likely combination of genotypes for the population at each position in the reference.

Positions of putatively polymorphic variants were identified and characterized by Variant Allele Frequency (VAF) - the fraction of variant alleles, out of all observed reads. Two types of biases were tested for each variant: Strand Bias (SB) is a type of sequencing bias in which one (positive/negative) strand is favored over the other, which can result in incorrect evaluation of the amount of evidence observed for one allele vs. the other. We used GATK Strand-Odds-Ratio (SOR) test [54] to determine if there is strand bias between forward and reverse strands for the reference or alternate alleles. Positions with SOR values > 4 may be considered as biased. Position Bias (PB) is a composite measure composed of (phred scaled) probabilities for observing positional bias within supporting reads for a variant. Positions with PB values > 40 may be considered as biased. Variants with VAF > 15% that passed SB (≤ 4) and PB (≤ 40) filters were marked as especially significant / strong.

#### Comparative genomics analysis of SNVs

All population sample variants genomes were taken from the NCBI GenBank [55]. Coordinates of the different genomic features (ORFs/UTRs) were obtained from DENV2 reference strain NC_001474.2 and aligned to the genomes used in this study. Multiple alignments of 618 DENV2 coding regions (i.e. DENVs population sample), and regions with conserved selection for strong or weak mRNA folding were computed as in [16]. In addition, we performed multiple alignments of all the 4 dengue serotypes combined with the Zika (population samples of 1672 and 332, respectively). Briefly, the translated *amino acid* sequences corresponding to the coding regions were aligned using the Clustal Omega package (version 1.2.0) with default parameters [56] and then mapped backed to the nucleotide sequences basing on the original nucleotide composition of each coding region. Multiple alignment conservation scores were defined as an average sum-of-pair score (SP; see Additional file 1, section 2). The accession numbers of the analyzed genomes can be found in Additional file 2.

Local minimum free folding energy (MFE) profiles were constructed by applying a 39 nt length sliding window to each genomic sequence: at each step the MFE of a local subsequence enclosed by the corresponding window was calculated by the Vienna (version 2.1.9) package RNAfold function with default parameters [57]. These profiles were compared to folding profiles of corresponding randomized variants preserving different genomic features of the wildtype sequences which are not necessarily directly related to mRNA folding: amino acid order and content, (“vertical”) distribution of synonymous codons at each position in the alignment, frequency of di-nucleotides. As a result, regions with positions that undergo a significantly conserved (across different genomes) selection at the synonymous/silent level for weak/strong mRNA folding (MFE-selected regions) were identified. More details, including the coordinates of the identified regions with significant folding signals, can be found in ref. [16] and in Additional file 1 (section 7).

Statistical analysis of the overlap between the positions of SNVs and regions selected for strong/weak RNA structure was performed as follows: from the analysis in [16] the total length of MFE - selected regions (for weak and strong folding) occupies ~ 1/3 of the entire coding sequence (3368/10176), i.e. a randomly chosen point lies in a MFE-selected region with a probability p ~ 1/3. On the other hand, in our case, 14 out of 32 SNV positions overlap with some MFE-selected region. In order to compare this number with what is expected in random under a Uniform distribution hypothesis we calculate the Hyper-geometric *p*-value: Let *N* (Population size) = 10,176 (total DENV2 CDS length); Successes in population (*K*) = 3368 (The total length of MFE - selected regions); Sample Size (n) = 32 (number of CDS SNV positions); Successes in Sample (*k*) = 14 (number of SNV positions that overlap with MFE-selected regions). Then, the probability to get at least 14 positions overlapping with MFE selected positions can be calculated as:

$$ p=1-\sum \limits_{k=1}^{13}\frac{\left(\begin{array}{l}K\\ {}k\end{array}\right)\left(\begin{array}{l}N-k\\ {}n-k\end{array}\right)}{\left(\begin{array}{l}N\\ {}n\end{array}\right)}\sim 0.14 $$

For a specific genomic position *i,* the nucleotide variability with respect to the population sample was quantified by normalized Shannon entropy of a distribution on nucleotides normalized by the maximal entropy value at this position in the corresponding alignment:

$$ {V}_i=\frac{\sum \limits_{j=1}^n{p}_j{\log}_2\left({p}_j\right)}{\log_2n} $$

Where *n* = 4, i.e. the number of different possible nucleotides. This variability measure takes values between 0 and 1, and describes how dispersed the distribution of the alphabet elements is: higher values correspond to more uniform nucleotide usage; lower values correspond to more biased nucleotide, indicating that some nucleotides are preferred/positions are conserved.

The level of significance (i.e. the *p*-value) was commonly determined based on the Wilcoxon rank sum test. The empirical p-value (reported in Fig. 4b), was determined based on a comparison between the average SNVs variability and the average variability set of non-SNV potions, with the same size (32 in this case).

Since the variability of the 3rd nucleotide in the codon is higher than of the other positions, the random sampling of the Non-SNVs positions was designed to have the same distribution as the SNVs. Specifically, we randomly selected Non-SNV values from the 1st/2nd/3rd positions in their codons, relatively to their frequencies; thus, all the randomly selected subsets of the Non-SNV has the same position distribution as the SNVs. In order to calculate the empirical *p*-values, we compared the average variability between the two sets (i.e. the SNV and the Non-SNV). The non-SNV set was chosen in random for 10,000 times and the p-value was determined as following: Let *S*_0_ be the value of the SNVs average and *S* = *S*_1_, *S*_2_, ⋯, *S*_*N*_ a vector containing *N* random values sampled from the non-SNV, where *S*_*i*_ is the value of the *i*_*th*_ sample; then $p=\frac{1}{N}\sum \limits_{i=1}^N\left|\left\{{\mathrm{S}}_i>{\mathrm{S}}_0\right\}\right|,N=10,000.$

#### Effective number of codons (ENC)

Is a measure that quantifies how far the codon usage of a coding sequence departs from equal usage of synonymous codons [58]; see more details in Additional file 1, section 3.

#### Codon pairs Bias (CPB)

To quantify codon pair bias, we follow [40] and define a codon pair score (CPS) as the log ratio of the observed over the expected number of occurrences of this codon pair in the coding sequence; see more details in Additional file 1, section 4.

#### Dinucleotide pair bias (DNTB), CpG content, and GC content

Following [59] we compute a dinucleotide score (DNTS) for a pair of nucleotides XY as an odds ratio of observed over expected frequencies; see more details in Additional file 1, sections 5–6.

### AG129 study

#### Animals

Male and female AG129 6–7 weeks old mice with an average weight of 20 g were used. Because of the immunocompromised nature of this strain, AG129 mice (produced in house) were housed in pre-sterilized bonneted (HEPA-filtered) cages in a ventilated cage rack during the duration of the experiment. Animals were randomly assigned to cages and individually marked with eartags. All instruments, eartags, gloved hands, or other surfaces that came in contact with the mice were cold-sterilized with a 10% betadine solution and/or 75% EtOH.

#### Facilities

Experiments were conducted in the BSL-2 animal suite at the Utah State University Laboratory Animal Research Center (LARC). All personnel receive continual special training on blood-borne pathogen handling by this university’s Environmental Health and Safety Office. Standard operating procedures for BSL-2 were used.

#### Quantification of viremia

The virus titers in plasma were assayed using the Brilliant II QRT-PCR Master Mix 1-step kit with samples run on the Mic (Magnetic Induction Cycler) real time PCR machine (Bio Molecular Systems, Inc). Briefly, RNA was extracted from serum samples collected 3 dpi using the QIAamp MinElute Virus Spin Kit (QIAGEN, cat# 57704), and was eluted with 25 μl of elution buffer. A volume of 5 μl of the RNA preparation was added to the appropriate mixture of PCR reagents following manufactures protocol. A virus stock of known titer was also extracted in parallel for use in quantification. Samples were subjected to 40 cycles of 15 s at 95 °C and 60 s at 60 °C following an initial single cycle of 30 min at 50 °C and 10 min at 95 °C. Samples of unknown quantity were quantified by extrapolation of C(t) values using a curve generated from serial dilutions of standard samples.

#### Experimental design

Animals were block-randomized by cage to two groups, with 8 included in each. Groups of 8 mice were challenged intraperitoneally (i.p.) with wild-type and synthetic viral variant. A third group of mice was also included as normal controls, and were sham-infected with vehicle to monitor handling, inoculation and caging techniques for effects on the immunocompromised AG129 mice. Mice were i.p. infected with 0.2 ml of each virus preparation. Mortality was observed daily for 28 days. Mice were weighed on day 0 and every other day through 11 dpi. Serum was collected from all animals on 3 dpi for quantification of viremia. Serum was also collected on 14 dpi for measurement of neutralizing antibody titer.

#### Statistical analysis of mice data

Survival data were analyzed using the Wilcoxon log-rank survival analysis, and all other statistical analyses were performed using one-way ANOVA with a Bonferroni group comparison.

#### Additional information

Animals were humanely euthanized when they met early euthanasia criteria. According to our institutionally approved protocol, animals unable to right themselves or displaying severe neurologic disease were humanely euthanized. Animals were observed at least twice daily throughout the study. Some deaths occur overnight, as occasionally animals will die without displaying severe signs of disease or will progress to mortality in the span of several hours. Since analgesics and anesthetics can potentially interfere with a viral infection, these methods were not utilized. Humane euthanasia is the primary mechanism to minimize animal suffering and distress. The animal research ethics committee at Utah State University approved the research with the approval # 2376. No anesthesia was used during the study and asphyxiation by CO_2_ inhalation was used for euthanasia at the end of the study.

## Additional files

Additional file 1: Supplementary Information:1 No difference in various genomic features in 100 codon regions around SNV compared to regions that do not contains SNVs. 2 Multiple alignments of the 618 DENV-2 genomes analyzed. 3 The Effective Number of Codons (ENC). 4 The Codon Pairs Bias (CPB). 5 The dinucleotide pair bias (DNTB). 6 CpG Content. 7 List of regions selected for strong/weak folding energy used. 8 Dengue virus type 2, New Guinea C master strain. (PDF 460 kb)

Additional file 2: List of the accession numbers of the DENV-1/DENV-2/DENV-3/DENV-4/ZIKV genomes analyzed. (XLSX 72 kb)
