# Supplementary material for: Generation and comparative genomics of synthetic dengue viruses
Source: BMC Bioinformatics. 2018 May 8;19(Suppl 6):140. doi: 10.1186/s12859-018-2132-3 (PMC5998877; doi:10.1186/s12859-018-2132-3)
Supplement: Supplementary file 1 — Supplementary Information:1 No difference in various genomic features in 100 codon regions around SNV compared to regions that do not contains SNVs. 2 Multiple alignments of the 618 DENV-2 genomes analyzed. 3 The Effective Number of Codons (ENC). 4 The Codon Pairs Bias (CPB). 5 The dinucleotide pair bias (DNTB). 6 CpG Content. 7 List of regions selected for strong/weak folding energy used. 8 Dengue virus type 2, New Guinea C master strain. (PDF 460 kb) [file 12859_2018_2132_MOESM1_ESM.pdf]

Generation and Comparative Genomics of Synthetic Dengue Viruses

Supplementary Information

Eli Goz[1,2,&], Yael Tsalenchuck[2,&], Rony Oren Benaroya[2], Zohar Zafrir[1,2], Shimshi Atar[1], Tahel Altman[2], Justin Julander[3], Tamir Tuller[1,2,4] *

[1] Department of Biomedical Engineering, Tel-Aviv University, Ramat Aviv, Israel. [2]SynVaccineLtd. Ramat Hachayal, Tel Aviv, Israel. [3] Institute for Antiviral Research, Utah State University, Logan, Utah, USA. [4]Sagol School of Neuroscience, Tel-Aviv University , Ramat Aviv, Israel.

& Equal contribution. *Corresponding author (TT): tamirtul@post.tau.ac.il

1. No difference in various genomic features in 100 codon regions around SNV compared to regions that do not contains SNVs

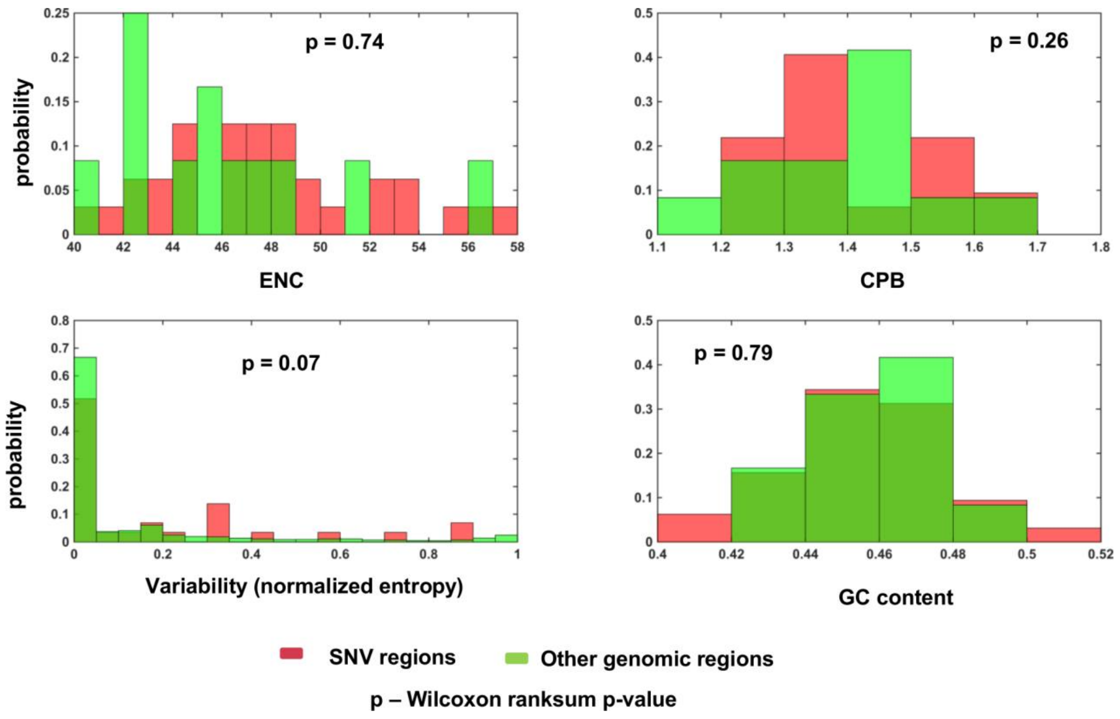

The results for two-sample Kolmogorov-Smirnov test were not significant as well; p = 0.96/0.19/0.3/0.99 for ENC/CPB/Variability/GC, respectively.

2. Multiple alignments of 618 DENV-2 genomes analyzed in this study - conservation scores

Multiple alignment conservation score was defined by us as an average sum-of-pair score (SP). For the i_{-th} column in the alignment we define $P_{ijk}=1$ for every pair A_{ij} and A_{ik} of elements (either nucleotides of amino acids, depending on the type of the

aligned sequences) which are equal to each other and $P_{ijk}=0$ otherwise. The score Si for the i_{th} column is

$$S_i = \frac{1}{N(N-1)/2} \sum_{j=1}^{N} \sum_{k=j+1}^{N} P_{ijk}$$

and the SP for the alignment is:

$$SP = \frac{1}{M} \sum_{i=1}^{M} S_i$$

The following values summarize the SP scores for the multiple alignment of 618 DENV-2 coding sequences analyzed in this study: SP(amino acids) = 0.97, SP(nucleotides) = 0.94.

3. The Effective Number of Codons (ENC)

The Effective Number of Codons (ENC) is a measure that quantifies how far the codon usage of a coding sequence departs from equal usage of synonymous codons. For each amino acid (AA) let us define x_i to be the number of its synonymous codons of each type in the sequence, and *n* to be the number of times this AA appears in the sequence:

$$n = \sum_{i}^{d} x_i$$

The frequency of each codon is therefore:

$$p_i = x_i / n$$

The ENC for a specific AA is:

$$ENC_A = 1 / F_A \text{ , where } F_A = \sum_{i}^{d} p_i^2$$

ENC for the group of AAs with degeneracy *d* (A_d):

$$ENC_{A_d} = 1 / F_{A_d} \text{ , where } F_{A_d} = \frac{1}{|A_d|} \sum_{A \in A_d} F_A$$

In case of a missing AA, the corresponding effective number of codons is defined as an average over the given AAs of the same degeneracy.

Finally ENC for a gene is defined as an average of the group ENCs over all the degeneracy AA groups weighted by the number of AAs in each group computed over the entire coding sequence.

$$ENC = 2 + \frac{9}{F_{A_2}} + \frac{1}{F_{A_3}} + \frac{5}{F_{A_4}} + \frac{3}{F_{A_6}}$$

ENC can take values from 20, in the case of extreme bias where one codon is exclusively used for each amino acid (AA), to 61 when the use of alternative synonymous codons is equally likely. Therefore smaller ENC values correspond to a higher bias in synonymous codons usage; consequently, a negative correlation with ENC values means is equivalent to a positive correlation with synonymous codons usage.

4. Codon Pairs Bias (CPB)

To quantify codon pair bias, we define a codon pair score (CPS) as the log ratio of the observed over the expected number of occurrences of this codon pair in the coding sequence. To achieve independence from amino acid and codon bias, the expected frequency is calculated based on the relative proportion of the number of times an amino acid is encoded by a specific codon:

$$CPS = \log\left(\frac{F(AB)}{\frac{F(A)\times F(B)}{F(X)\times F(Y)}\times F(XY)}\right),$$

where the codon pair AB encodes for amino acid pair XY and F denotes the number of occurrences. The codon pair bias (CPB) of a virus is then defined as an average of codon pair scores over all codon pairs comprising all viral coding sequences:

$$CPB = \frac{1}{k-1}\sum_{i=1}^{k-1} CPS[i]$$

5. The dinucleotide pair bias (DNTB)

The dinucleotide pair bias (DNTB) of a virus is defined as an average of dinucleotide scores over all dinucleotides comprising all viral sequences:

$$DNTB = \frac{1}{k-1}\sum_{i=1}^{k-1} DNTS[i]$$

The **GC content** is defined as:

$$GC\% = \frac{F(G)+F(C)}{F(A)+F(G)+F(C)+F(T)}$$

Where *F()* is a number of occurrences of each one of nucleotides A,G,C, and T.

6. CpG Content

We compute a dinucleotide score (DNTS) for a pair of nucleotides XY as an odds ratio of observed over expected frequencies:

$$DNTS = \frac{F(XY)}{F(X)F(Y)},$$

where *F* denotes the frequency of occurrences.

Specifically, the CpG score is equal to the DNTS corresponding to the CG nucleotide.

7. List of regions selected for strong/weak folding energy used in this study

Coordinates of regions predicted to be selected for strong/weak folding energy can be found in the following tables (see details in reference [16] in main text):

Each row in a file corresponds to one region (number of rows = number of regions) and contains 3 comma separated values x, y, z in the following order:

region start coordinate, region end coordinate, maximum folding selection conservation index (FSCI) in the cluster.

The coordinates are given with respect to the start of the polyprotein coding sequence in the reference genome NC_001474.2

E.g., coordinates x, y for some region correspond to the nucleotides at x_{th} and y_{th} positions in the coding sequence of NC_001474.2

weak folding		
start	end	FSCI
96	153	0.47
186	247	0.33
332	416	0.43
441	490	0.23
530	686	0.93
781	830	0.29
867	917	0.31
1098	1191	0.95
1263	1311	0.49
1333	1381	0.33
1506	1563	0.3
1617	1673	0.4
1705	1840	0.66
1879	1968	0.82

strong folding		
119	165	0.21
266	353	0.29
387	615	0.74
695	745	0.44
820	872	0.68
1010	1142	0.48
1179	1272	0.31
1287	1332	0.21
1353	1520	0.74
1521	1583	0.54
1585	1635	0.57
1662	1739	0.85
1964	2078	0.48
2157	2231	0.43
2244	2313	0.36

2020	2086	0.66
2123	2171	0.54
2219	2291	0.49
2374	2432	0.28
2817	2865	0.33
2885	2933	0.33
2998	3061	0.38
3062	3107	0.22
3258	3328	0.32
3378	3438	0.38
3564	3613	0.56
3717	3766	0.35
3844	3904	0.34
4046	4106	0.74
4204	4292	0.53
4355	4403	0.32
4465	4563	0.65
4576	4625	0.25
4629	4682	0.41
4758	4806	0.28
5028	5077	0.26
5239	5290	0.21
5299	5387	0.36
5458	5557	0.45
5626	5674	0.23
5772	5823	0.68
5844	5940	0.62
6129	6179	0.3
6186	6231	0.71
6338	6386	0.24
6475	6589	0.8
6649	6718	0.38
6752	6815	0.29
6895	6983	0.45
6995	7183	0.93
7240	7299	0.65
7332	7379	0.26
7412	7461	0.3
7658	7709	0.32
7731	7780	0.3
7785	7835	0.5
7913	8004	0.29
8022	8079	0.5
8207	8266	0.3
8279	8331	0.51
8409	8463	0.67
8470	8556	0.63

2345	2413	0.37
2523	2688	0.7
2689	2805	0.48
2839	2903	0.38
2932	2980	0.26
2991	3038	0.2
3263	3312	0.51
3333	3406	0.8
3419	3464	0.27
3481	3545	0.41
3572	3631	0.35
3793	3838	0.2
3845	3941	0.21
3972	4020	0.33
4029	4080	0.35
4122	4171	0.29
4197	4251	0.32
4347	4399	0.35
4407	4525	0.52
4680	4732	0.44
4869	4914	0.21
5083	5131	0.24
5142	5235	0.87
5347	5396	0.22
5567	5618	0.5
5940	6000	0.48
6080	6150	0.78
6230	6282	0.31
6328	6376	0.26
6712	6776	0.48
6803	6871	0.42
6946	6994	0.37
7137	7187	0.34
7192	7271	0.52
7286	7434	0.68
7608	7668	0.63
7679	7727	0.25
7799	7851	0.25
7871	7928	0.45
8073	8166	0.49
8229	8296	0.41
8345	8393	0.27
8696	8754	0.41
8873	8951	0.98
9258	9309	0.47
9460	9508	0.23
9585	9636	0.67

8580	8624	0.25
8747	8821	0.49
8920	8969	0.34
9046	9102	0.6
9156	9213	0.53
9214	9292	0.94
9344	9385	0.2
9395	9532	0.78
9578	9691	0.74
9829	9887	0.56
10019	10069	0.3
10109	10167	0.46

9666	9848	0.95
9880	9944	0.57
9950	10021	0.51

8. Dengue virus type 2, New Guinea C master strain (GenBank accession: KM204118.1) – nucleotide sequence

AGTTGTTAGTCTACGTGGACCGACAAAGACAGATTCTTTGAGGGAGCTAA

GCTCAACGTAGTTCTAACAGTTTTTTAATTAGAGAGCAGATCTCTGATGA

ATAACCAACGAAAAAAGGCGAGAAATACGCCTTTCAATATGCTGAAACGC

GAGAGAAACCGCGTGTCGACTGTACAACAGCTGACAAAGAGATTCTCACT

TGGAATGCTGCAGGGACGAGGACCATTAAAACTGTTCATGGCCCTGGTGG

CGTTCCTTCGTTTCCTAACAATCCCACCAACAGCAGGGATACTGAAGAGA

TGGGGAACAATTAAAAAATCAAAAGCCATTAATGTTTTGAGAGGGTTCAG

GAAAGAGATTGGAAGGATGCTGAACATCTTGAACAGGAGACGCAGAACTG

CAGGCATGATCATTATGCTGATTCCAACAGTGATGGCGTTCCATTTAACC

ACACGTAACGGAGAACCACACATGATCGTCAGTAGACAAGAGAAAGGGAA

AAGTCTTCTGTTTAAAACAGAGGATGGTGTGAACATGTGTACCCTCATGG

CCATGGACCTTGGTGAATTGTGTGAAGATACAATCACGTACAAGTGTCCT

CTTCTCAGGCAGAATGAACCAGAAGACATAGATTGTTGGTGCAACTCTAC

GTCCACATGGGTAACTTATGGGACGTGTACCACCACAGGAGAACACAGAA

GAGAAAAAAGATCAGTGGCACTCGTTCCACATGTGGGAATGGGACTGGAG

ACACGAACTGAAACATGGATGTCATCAGAAGGGGCCTGGAAACATGCCCA

GAGAATTGAAACTTGGATCTTGAGACATCCAGGCTTTACCATAATGGCAG

CAATCCTGGCATACACCATAGGAACGACACATTTCCAAAGAGCCCTGATT

TTCATCTTACTGACAGCTGTCGCTCCTTCAATGACAATGCGTTGCATAGG

AATATCAAATAGAGACTTTGTAGAAGGGGTTTCAGGAGGAAGCTGGGTTG

ACATAGTCTTAGAACATGGAAGCTGTGTGACGACGATGGCAAAAAACAAA

CCAACATTGGATTTTGAACTGATAAAAACAGAAGCCAAACAACCTGCCAC

TCTAAGGAAGTACTGTATAGAGGCAAAGCTGACCAACACAACAACAGAAT

CTCGCTGCCCAACACAAGGAGAACCCAGCCTAAATGAAGAGCAGGACAAA

AGGTTCGTCTGCAAACACTCCATGGTGGACAGAGGATGGGGAAATGGATG

TGGATTATTTGGAAAAGGAAGCATTGTGACCTGTGCTATGTTCACATGCA

AAAAGATCATGAAAGGAAAAGTCGTGCAACCAGAAAACTTGGAATACACC

ATTGTGATAACACCTCACTCAGGGGAAGAGCATGCAGTCGGAAATGACAC

AGGAAAACATGGCAAGGAAATCAAAATAACACCACAGAGTTCCATCACAG

AAGCAGAGTTGACAGGCTATGGCACTGTCACGATGGAGTGCTCTCCGAGA

ACGGGCCTCGACTTCAATGAGATGGTGTTGCTGCAAATGGAAAATAAAGC

TTGGCTGGTGCACAGGCAATGGTTCCTAGACCTGCCGTTGCCATGGCTGC

CCGGAGCGGACACACAAGGATCAAATTGGATACAGAAAGAGACATTGGTC

ACTTTCAAAAATCCCCATGCGAAGAAACAGGATGTTGTTGTTTTGGGATC

CCAAGAAGGGGCCATGCACACAGCACTCACAGGGGCCACAGAAATCCAGA

TGTCATCAGGAAACTTACTGTTCACAGGACATCTCAAGTGCAGGCTGAGG

ATGGACAAACTACAGCTCAAAGGAATGTCATACTCTATGTGCACAGGAAA

GTTTAAAGTTGTGAAGGAAATAGCAGAAACACAACATGGAACAATAGTTA

TCAGAGTACAATATGAAGGGGACGGTTCTCCATGCAAGATCCCTTTTGAG

ATAATGGATTTGGAAAAAAGACATGTTTTAGGTCGCCTGATTACAGTCAA

CCCAATCGTAACAGAAAAAGATAGCCCAGTCAACATAGAAGCAGAACCTC

CATTCGGAGACAGCTACATCATCATAGGAGTAGAGCCGGGACAATTGAAG

CTCAACTGGTTTAAGAAAGGAAGTTCTATCGGCCAAATGTTTGAGACAAC

AATGAGGGGAGCGAAGAGAATGGCCATTTTAGGTGACACAGCTTGGGATT

TTGGATCCCTGGGAGGAGTGTTTACATCTATAGGAAAGGCTCTCCACCAA

GTTTTCGGAGCAATCTATGGGGCTGCCTTCAGTGGGGTCTCATGGACTAT

GAAAATCCTCATAGGAGTCATTATCACATGGATAGGAATGAATTCACGCA

GCACCTCACTGTCTGTGTCACTAGTATTGGTGGGAGTCGTGACGCTGTAT

TTGGGAGTTATGGTGCAGGCCGATAGTGGTTGCGTTGTGAGCTGGAAAAA

CAAAGAACTGAAGTGTGGCAGTGGGATTTTCATCACAGACAACGTGCACA

CATGGACAGAACAATACAAGTTCCAACCAGAATCCCCTTCAAAACTAGCT

TCAGCTATCCAGAAAGCTCATGAAGAGGGCATTTGTGGAATCCGCTCAGT

AACAAGACTGGAAAATCTGATGTGGAAACAAATAACACCAGAATTGAATC

ACATTCTATCAGAAAATGAGGTGAAGTTGACTATTATGACAGGAGACATC

AAAGGAATCATGCAGGCAGGAAAACGATCTCTGCGGCCCCAGCCCACTGA

GCTGAAGTATTCATGGAAAACATGGGGCAAAGCGAAAATGCTCTCTACAG

AGTCTCATAACCAGACCTTTCTCATTGATGGCCCCGAAACAGCAGAATGC

CCCAACACAAACAGAGCTTGGAATTCGCTGGAAGTTGAAGACTATGGCTT

TGGAGTATTCACCACCAATATATGGCTAAAGTTGAGAGAAGAGCAGGATG

TATTCTGCGACTCAAAACTCATGTCAGCGGCCATAAAAGACAACAGAGCC

GTCCATGCCGATATGGGTTATTGGATAGAAAGTGCACTCAATGACACATG

GAAGATAGAGAAAGCCTCTTTCATCGAAGTTAAAAGCTGCCACTGGCCAA

AGTCACACACCCTCTGGAGTAATGGAGTGCTAGAAAGTGAGATGATAATT

CCAAAGAATTTCGCTGGACCAGTGTCACAACACAACTACAGACCAGGCTA

CCATACACAAACAGCAGGACCATGGCATCTAGGTAAGCTTGAGATGGACT

TTGATTTCTGCGAAGGAACCACAGTGGTGGTGACTGAGGACTGTGGAAAT

AGAGGACCCTCTTTAAGAACAACTACTGCCTCTGGAAAACTCATAACAGA

ATGGTGCTGCCGATCTTGCACATTACCACCGCTAAGATACAGAGGTGAGG

ACGGATGCTGGTACGGGATGGAAATCAGACCATTGAAAGAGAAAGAAGAG

AATTTGGTCAACTCCTTGGTCACAGCCGGACATGGGCAGATTGACAACTT

TTCACTAGGAGTCTTGGGAATGGCATTGTTCCTGGAAGAAATGCTCAGGA

CCCGAGTAGGAACGAAACATGCAATACTACTAGTTGCAGTTTCTTTTGTG

ACATTGATCACAGGGAACATGTCCTTTAGAGACCTGGGAAGAGTGATGGT

TATGGTGGGCGCTACTATGACGGATGACATAGGTATGGGCGTGACTTATC

TTGCCCTCCTAGCAGCCTTCAAAGTCAGACCAACTTTTGCAGCTGGACTA

CTCTTGAGAAAGTTGACCTCCAAGGAATTGATGATGACTACCATAGGAAT

CGTACTCCTCTCCCAGAGCACCATACCAGAGACCATTCTTGAACTGACTG

ATGCGTTAGCCTTGGGCATGATGGTCCTTAAAATGGTGAGAAAAATGGAA

AAGTATCAATTGGCAGTGACTATCATGGCTATCTTGTGCGTCCCAAATGC

AGTGATATTACAAAACGCATGGAAAGTGAGTTGCACAATATTGGCAGTGG

TGTCCGTTTCCCCACTGTTCTTAACATCCTCACAGCAGAAAGCGGATTGG

ATACCATTAGCATTGACGATCAAGGGTCTCAATCCAACAGCTATTTTTCT

AACAACCCTTTCAAGAACCAACAAGAAAAGGAGCTGGCCACTAAATGAGG

CTATCATGGCAGTCGGGATGGTGAGCATTTTGGCCAGTTCACTCCTAAAG

AATGACATTCCCATGACAGGACCATTAGTGGCTGGAGGGCTCCTCACTGT

GTGCTACGTGCTCACTGGACGATCGGCCGATTTGGAACTGGAGAGAGCCG

CCGATGTCAAATGGGAAGATCAGGCAGAGATATCAGGAAGCAGTCCAATC

CTGTCAATAACAATATCAGAAGATGGTAGCATGTCGATAAAAAACGAAGA

GGAAGAACAAACACTGACCATACTCATTAGAACAGGATTGCTGGTGATCT

CAGGACTTTTTCCTGTATCAATACCAATCACGGCAGCAGCATGGTACCTG

TGGGAAGTGAAGAAACAACGGGCTGGAGTATTGTGGGATGTCCCTTCACC

CCCACCCGTGGGAAAGGCTGAACTGGAAGATGGAGCCTATAGAATCAAGC

AAAAAGGGATTCTTGGATATTCCCAGATCGGAGCCGGAGTTTACAAAGAA

GGAACATTCCATACAATGTGGCATGTCACACGCGGCGCTGTTCTAATGCA

TAAAGGAAAGAGGATTGAACCATCATGGGCGGACGTTAAGAAAGACCTAA

TATCATATGGAGGAGGCTGGAAGCTAGAAGGAGAATGGAAGGAAGGAGAA

GAAGTCCAGGTCTTGGCATTGGAGCCTGGAAAAAATCCAAGAGCCGTCCA

AACAAAACCTGGTCTTTTCAAAACCAACGCCGGAACCATAGGTGCCGTAT

CTCTGGACTTTTCTCCTGGAACCTCAGGATCTCCAATCATCGACAAAAAA

GGAAAAGTTGTGGGTCTTTATGGTAATGGTGTTGTTACAAGGAGTGGAGC

ATATGTGAGTGCTATAGCCCAGACTGAAAAAAGTATTGAAGACAATCCAG

AGATCGAAGACGACATTTTTCGAAAGAGAAAATTGACCATCATGGACCTC

CACCCAGGAGCGGGAAAGACGAAGAGATACCTTCCGGCCATAGTCAGAGA

GGCTATAAAACGGGGCCTGAGGACATTAATCCTGGCCCCCACTAGAGTCG

TGGCAGCTGAAATGGAGGAAGCCCTAAGAGGACTTCCAATAAGATACCAA

ACCCCAGCCATCAGAGCTGAGCACACCGGGCGGGAGATTGTGGACCTAAT

GTGTCATGCCACATTCACTATGAGGCTGCTATCACCAGTTAGAGTGCCAA

ATTACAACCTGATCATCATGGACGAAGCCCATTTCACAGACCCAGCAAGT

ATAGCGGCTAGAGGATACATCTCAACTCGAGTAGAGATGGGTGAGGCAGC

TGGGATTTTCATGACAGCCACTCCTCCGGGAAGCAGAGACCCATTCCCTC

AGAGCAATGCACCAATCATGGATGAAGAAAGAGAAATCCCTGAACGTTCG

TGGAGTTCTGGACATGAGTGGGTCACGGATTTTAAAGGGAAGACTGTTTG

GTTCGTTCCAAGTATAAAAGCAGGAAATGATATAGCAGCTTGCCTGAGAA

AAAATGGAAAGAAAGTGATACAACTCAGTAGGAAGACCTTTGATTCTGAG

TATGTCAAGACTAGAACCAATGATTGGGACTTCGTGGTCACAACTGACAT

TTCAGAAATGGGTGCCAACTTCAAGGCTGAGAGGGTTATAGACCCCAGAC

GCTGCATGAAACCAGTTATACTAACAGATGGTGAAGAGCGGGTGACCCTG

GCAGGACCTATGCCAGTGACCCACTCTAGTGCAGCACAAAGAAGAGGGAG

AATAGGAAGAAATCCAAAAAATGAAAATGACCAGTACATATACATGGGGG

AACCTCTGGAAAATGATGAAGACTGTGCACACTGGAAAGAAGCTAAAATG

CTCCTAGATAACATCAACACACCTGAAGGAATCATTCCTAGCATGTTCGA

ACCAGAGCGTGAAAAGGTGGATGCCATTGATGGTGAATACCGCTTGAGAG

GAGAAGCAAGGAAAACCTTTGTGGACCTAATGAGAAGAGGAGACCTACCA

GTCTGGTTGGCCTACAGAGTGGCAGCTGAAGGCATCAACTACGCAGACAG

AAGGTGGTGTTTTGATGGAATTAAGAACAACCAAATCTTGGAAGAAAATG

TGGAGGTGGAAATCTGGACAAAAGAAGGGGAAAGGAAGAAATTAAAACCC

AGATGGTTGGATGCCAGGATCTACTCTGACCCACTGGCGCTAAAGGAATT

CAAGGAGTTTGCAGCTGGAAGAAAGTCCCTGACCCTGAACCTAATCACAG

AAATGGGTAGGCTTCCAACTTTCATGACTCAGAAGGCAAGAGACGCACTG

GACAACTTAGCAGTGCTGCACACGGCTGAAGCAGGTGGAAGGGCGTACAA

TCATGCTCTCAGTGAACTGCCGGAGACCCTGGAGACATTGCTTTTACTGA

CACTTCTGGCTACAGTCACGGGAGGAATCTTTTTATTCTTGATGAGCGGA

AGGGGTATAGGGAAGATGACCCTGGGAATGTGCTGCATAATCACGGCTAG

TATTCTCCTATGGTACGCACAAATACAGCCACACTGGATAGCAGCTTCAA

TAATACTGGAGTTTTTTCTCATAGTTTTGCTTATTCCAGAACCAGAAAAG

CAGAGAACACCCCAAGATAACCAATTGACCTACGTTGTCATAGCCATCCT

CACAGTGGTGGCCGCAACCATGGCAAACGAGATGGGTTTCCTGGAAAAAA

CGAAGAAAGATCTCGGATTGGGAAGCATTACAACCCAGCAACCCGAGAGC

AACATCCTGGACATAGATCTACGTCCCGCATCAGCATGGACGCTGTATGC

TGTGGCCACAACATTTGTCACACCAATGTTAAGACACAGCATTGAAAATT

CCTCAGTGAACGTGTCCCTAACAGCTATTGCCAACCAAGCCACAGTGTTA

ATGGGTCTTGGGAAAGGATGGCCATTGTCAAAGATGGACATCGGAGTTCC

CCTTCTCGCCATTGGATGCTACTCACAAGTCAACCCCATAACTCTCACAG

CAGCTCTTTTCTTACTGGTAGCACATTATGCCATCATAGGGCCAGGACTC

CAAGCAAAAGCAACCAGAGAAGCTCAGAAAAGAGCAGCAGCGGGCATCAT

GAAAAACCCAACTGTCGATGGAATAACAGTGATTGACCTAGATCCAATAC

CCTATGATCCAAAGTTTGAAAAGCAGTTGGGACAAGTAATGCTCCTAGTC

CTCTGCGTGACTCAAGTGTTGATGATGAGGACTACATGGGCTCTGTGTGA

GGCTTTAACCTTAGCGACCGGGCCTATCTCCACATTGTGGGAAGGAAATC

CAGGGAGGTTTTGGAACACTACCATTGCAGTGTCAATGGCTAACATTTTT

AGAGGGAGTTACTTGGCCGGAGCTGGACTTCTCTTTTCCATCATGAAGAA

CACAACCAACACGAGAAGGGGAACTGGCAACATAGGAGAGACGCTTGGAG

AGAAATGGAAAAGCCGATTGAACGCATTGGGGAAAAGTGAATTCCAGATC

TACAAGAAAAGTGGAATCCAGGAAGTGGATAGAACCTTAGCAAAAGAAGG

CATTAAAAGAGGAGAAACGGACCATCACGCTGTGTCGCGAGGCTCAGCAA

AACTGAGATGGTTCGTCGAGAGAAATATGGTCACACCAGAAGGGAAAGTA

GTGGACCTCGGTTGCGGCAGAGGAGGCTGGTCATACTATTGTGGGGGACT

AAAGAATGTAAGAGAAGTCAAAGGCCTAACAAAAGGAGGACCAGGACATG

AAGAACCCATCCCCATGTCAACATATGGGTGGAATCTAGTACGTCTTCAA

AGTGGAGTTGACGTTTTCTTCACTCCGCCAGAAAAGTGTGACACATTGTT

GTGTGACATAGGGGAGTCGTCACCAAATCCCACGGTAGAAGCAGGACGAA

CACTCAGAGTCCTTAACTTAGTGGAAAATTGGTTGAACAACAACACCCAA

TTTTGCATAAAGGTTCTCAACCCATACATGCCCTCAGTCATAGAAAAAAT

GGAAGCACTACAAAGGAAATATGGAGGAGCCTTAGTGAGGAATCCACTCT

CACGAAACTCCACACATGAGATGTACTGGGTATCCAATGCCTCCGGGAAC

ATAGTGTCATCAGTGAACATGATTTCAAGGATGTTGATCAACAGATTCAC

AATGAGACACAAGAAAGCCACTTACGAGCCAGATGTAGACCTCGGAAGCG

GAACCCGCAACATCGGAATTGAAAGTGAGATACCAAACCTAGACATAATC

GGGAAAAGAATAGAAAAAATAAAACAAGAGCATGAAACATCATGGCACTA

TGACCAAGACCACCCATACAAAACGTGGGCTTACCATGGCAGCTATGAAA

CAAAACAAACTGGATCAGCATCATCCATGGTGAACGGAGTGGTCAGACTG

CTGACAAAACCTTGGGACGTCGTCCCCATGGTGACACAGATGGCAATGAC

AGACACGACTCCATTTGGACAACAGCGCGTTTTTAAAGAAAAAGTGGACA

CGAGAACCCAAGAACCGAAAGAAGGCACAAAGAAACTAATGAAAATCACG

GCAGAGTGGCTTTGGAAAGAACTAGGGAAGAAAAAGACACCTAGGATGTG

CACTAGAGAAGAATTCACAAGAAAGGTGAGAAGCAATGCAGCCTTGGGGG

CCATATTCACTGATGAGAACAAGTGGAAGTCGGCACGTGAGGCTGTTGAA

GATAGTAGGTTTTGGGAGCTGGTTGACAAGGAAAGGAATCTCCATCTTGA

AGGAAAGTGTGAAACATGTGTGTATAACATGATGGGAAAAAGAGAGAAGA

AGCTAGGGGAGTTCGGCAAGGCAAAAGGCAGCAGAGCCATATGGTACATG

TGGCTTGGAGCACGCTTCTTAGAGTTTGAAGCCCTAGGATTCTTGAATGA

AGATCACTGGTTCTCCAGAGAGAACTCCTTGAGTGGAGTGGAAGGAGAAG

GGCTGCACAAGCTAGGTTACATTTTAAGAGACGTGAGCAAGAAAGAGGGA

GGAGCAATGTATGCCGATGACACCGCAGGATGGGACACAAGAATCACACT

AGAAGACCTAAAAAATGAAGAAATGGTAACAAACCACATGGAAGGAGAAC

ACAAGAAACTAGCCGAGGCCATTTTCAAATTAACGTACCAAAACAAGGTG

GTGCGTGTGCAAAGACCAACACCAAGAGGCACAGTAATGGATATCATATC

GAGAAGGGACCAAAGAGGTAGTGGACAAGTTGGTACCTATGGACTCAATA

CTTTCACCAATATGGAAGCCCAACTAATCAGACAGATGGAGGGAGAAGGA

GTCTTCAAAAGCATTCAGCACCTGACAGTCACAGAAGAAATCGCCGTGCA

AAACTGGTTAGCAAGAGTAGGGCGCGAAAGGTTATCAAGAATGGCCATCA

GTGGAGATGATTGTGTTGTGAAACCTTTAGATGACAGGTTCGCAAGCGCT

TTAACAGCTCTAAATGACATGGGAAAGGTTAGGAAAGACATACAACAATG

GGAACCTTCAAGAGGATGGAACGATTGGACACAAGTGCCCTTCTGTTCAC

ACCATTTCCATGAGTTAATCATGAAAGACGGCCGCGTACTTGTAGTTCCA

TGCAGAAACCAAGATGAACTGATTGGTAGAGCCCGAATTTCCCAAGGAGC

TGGGTGGTCTTTGCGAGAGACGGCCTGTTTGGGGAAGTCCTACGCCCAAA

TGTGGAGCTTGATGTACTTCCACAGACGTGACCTCAGGCTGGCGGCTAAT

GCTATTTGCTCGGCAGTCCCATCACATTGGGTTCCAACAAGTAGAACAAC

CTGGTCCATACACGCCAAACATGAATGGATGACAACGGAAGACATGCTGA

CAGTCTGGAACAGGGTGTGGATTCAAGAAAACCCATGGATGGAAGACAAA

ACTCCAGTGGAATCATGGGAGGAAATCCCATACTTGGGGAAAAGAGAAGA

CCAATGGTGCGGCTCATTGATTGGGCTAACAAGCAGGGCCACCTGGGCAA

AGAACATCCAAACAGCAATAAATCAAGTTAGATCCCTTATAGGCAATGAG

GAATACACAGATTACATGCCATCCATGAAAAGATTCAGAAGAGAAGAGGA

AGAGGCAGGAGTCCTGTGGTAGAAGGCAAAACTAACATGAAACAAGGCTA

GAAGTCAGGTCGGATTAAGCCATAGTACGGAAAAAACTATGCTACCTGTG

AGCCCCGTCCAAGGACGTTAAAAGAAGTCAGGCCATTACAAATGCCATAG

CTTGAGTAAACTGTGCAGCCTGTAGCTCCACCTGAGAAGGTGTAAAAAAT

CTGGGAGGCCACAAACCATGGAAGCTGTACGCATGGCGTAGTGGACTAGC

GGTTAGAGGAGACCCCTCCCTTACAAATCGCAGCAACAATGGGGGCCCAA

GGTGAGATGAAGCTGTAGTCTCACTGGAAGGACTAGAGGTTAGAGGAGAC

CCCCCCAAAACAAAAAACAGCATATTGACGCTGGGAAAGACCAGAGATCC

TGCTGTCTCCTCAGCATCATTCCAGGCACAGAACGCCAGAAAATGGAATG

GTGCTGTTGAATCAACAGGTTCT
